# Research on multi-objective emergency resource scheduling optimization in chemical industrial parks

**DOI:** 10.1371/journal.pone.0332858

**Published:** 2025-09-30

**Authors:** Yuhang Wang, Mingguang Zhang, Jun Lu, Yufei Gui

**Affiliations:** 1 College of Safety Science and Engineering, Nanjing Tech University, Nanjing, Jiangsu, China; 2 Jiangsu Key Laboratory of Hazardous Chemicals Safety and Control, Nanjing, Jiangsu, China; Beijing University of Technology, CHINA

## Abstract

The high concentration of hazardous sources in chemical parks, which is prone to cause chain accidents, puts forward the demand for dynamic cooperative optimization of emergency resource scheduling. Aiming at the deficiencies of existing studies in the adaptability of dynamic multi-hazard scenarios and the quantification of resource allocation fairness, this paper constructs a three-objective mixed-integer planning model that integrates time efficiency, demand coverage and allocation fairness. Fairness is innovatively quantified as an independent optimization objective, and a standard deviation-based dynamic resource allocation balance index is proposed, which combines multi-warehouse collaborative supply and multi-resource coupling constraint mechanism to systematically solve the problem of trade-offs between timeliness, adequacy and fairness in emergency dispatching in chemical accidents. The improved NSGA-II algorithm is used to solve the Pareto front efficiently, and the search efficiency is improved by the elite reservation strategy and the congestion adaptive adjustment mechanism. In the case study, comparative experiments with the weighted method and the MOGWO algorithm demonstrate that NSGA-II performs superiorly in key metrics, exhibiting excellent convergence, diversity, and stability. Based on this, a case study is conducted using a chemical industrial park in China as an example, generating 41 sets of weights covering extreme preferences, two-objective balance, and three-objective balance. Decision-makers screen solutions based on loss tolerance thresholds and select the optimal solution using a composite score of comprehensive weighted losses. The study further reveals that improvements in demand satisfaction rates are often accompanied by significant increases in transportation time, while pursuing optimal fairness may weaken overall demand satisfaction levels. Sensitivity analysis confirms that resource demand is the key driver determining the number of feasible solutions, while fairness, as an independent optimization objective, holds irreplaceable importance in emergency scheduling decisions.

## 1. Introduction

The chemical industry has become a cornerstone of the national economic system, with chemical enterprises increasingly pursuing intensive development and gradually establishing chemical industrial parks, resulting in a growing demand for enhanced emergency response capabilities. Within chemical parks, a variety of production and storage facilities are densely distributed, and a large number of flammable, explosive, or toxic hazardous chemicals are widely used in daily production management. This makes the sources of danger highly concentrated in the park, making it prone to domino accidents that can cause widespread damage. Moreover, as a cluster of enterprises, chemical parks are densely populated, and once they are affected by major natural disasters such as earthquakes and floods, many enterprises within the park are inevitably damaged to varying degrees, and may even trigger chain reactions in multiple areas, further expanding the disaster situation. The large-scale accidents caused by various factors may lead to the inability to implement emergency resource scheduling plans in a timely manner, resulting in a series of problems such as resource waste, chaotic rescue processes, and low efficiency in the operation of the rescue site. Therefore, it is of great significance to carry out research on the optimization of emergency resource scheduling for multiple disaster points in chemical parks.

### 1.1 Research on emergency resource scheduling optimization

The core issue in emergency resource dispatching is how to quickly design an effective resource allocation plan after an accident occurs, with the aim of minimizing casualties caused by the incident. Post-disaster resource scheduling is mainly achieved by constructing a mathematical model that includes constraints and an objective function. Such models, while satisfying a series of constraints, can scientifically and reasonably deliver resources to the affected areas and seek the optimal solution for the objective function among feasible plans.

#### 1.1.1 Single-objective research.

Traditional single-objective emergency resource scheduling research focuses on minimizing transport time [1,2] or cost [3] in priority and mainly relies on deterministic methods such as linear programming [4]. Although such models are computationally efficient in small-scale static scenarios, their core shortcoming is that they grossly neglect the need for differential prioritization in multi-disaster regions, leading to significant limitations in their adaptability in real-world scenarios such as complex chemical accidents. More fundamentally, these models are inherently incapable of addressing the complex trade-offs between time, cost and fairness in resource allocation. It is this inherent limitation that directly drives the development of multi-objective optimization methods in the field of emergency resource scheduling.

#### 1.1.2 Multi-objective research.

Although single-objective optimization is relatively mature in the field of emergency resource scheduling, the academic community is actively turning to multi-objective optimization models in order to better fit the complex reality of post-disaster situations. The core challenge of such models is to weigh conflicting objectives and effectively incorporate realistic constraints [5]. Current research is advancing in three directions:

Dynamic and Uncertainty Modelling: Research is gradually moving beyond static assumptions and attempting to integrate time-varying demands [6]and probabilistic scenarios [7], the integration of pre-disaster prevention [8] and post-disaster relief decisions [9] in earthquake response decision-making. However, such integration often relies on specific probability distribution assumptions or a limited set of scenarios, and is still weak in dealing with extreme events or deep uncertainty, and the robustness of the model is questionable.

Complex Systems Integration: Efforts have been made to incorporate location-path co-optimization [10], multi-resource coupling constraints [11], and even psychological behavioral factors of disaster victims/rescuers into a unified framework [12]. While such integration attempts are certainly worthy of recognition, they often lead to highly complex models with numerous and difficult-to-access parameters, and face great challenges in terms of practical operability and accurate quantification of coupling effects among different modules, and there is a significant gap between theory and practice.

Algorithmic Innovations and Applications: Given the complexity of multi-objective problems, evolutionary algorithms [13] and their hybrid variants [14–16] are widely used to solve large-scale problems. Studies claim that they are superior to traditional weighted sum methods in terms of solution diversity and computational efficiency [17,18]. However, the superiority of these algorithms is mostly verified on specific arithmetic examples or simplified models, and their stability and convergence speed in large-scale, high-dimensional, and strongly constrained real emergency response scenarios still need more empirical tests. The ‘black-box’ nature of the algorithms sometimes undermines the decision makers’ understanding and trust in the solutions.

In summary, there are two critical and interrelated shortcomings in the current research. In terms of significant domain specificity, the vast majority of model development and validation has been overly focused on natural hazards. This focus results in models that may fail in their predefined response patterns, resource requirements and constraints when responding to man-made disasters or complex crises. The lack of a generalized framework or adaptive mechanism across disaster types is a significant barrier to the wider application of models. Secondly, in terms of the ambiguity and absence of fairness objectives, although ‘fairness’ is sometimes mentioned lightly or embedded as a soft constraint, there is a paucity of research that explicitly defines it as a core optimization objective and establishes effective quantitative criteria, especially in scenarios involving competing resource demands across multiple disaster sites. Equity is often masked or simplified by efficiency or cost objectives, and there is a lack of in-depth consideration of social justice and ethical dimensions and actionable quantitative modelling. This absence not only weakens the relevance of the models, but may also lead to ethically inappropriate optimization results.

### 1.2 Multi-objective optimization solution method research

Multi-Objective Optimization is a field that deals with problems where multiple conflicting or competing objective functions exist simultaneously [19]. When addressing such problems, optimizing one objective function may lead to a decline in the performance of others. Therefore, multi-objective optimization problems do not have a single optimal solution but rather a set of best solutions that satisfy multiple objectives, known as Pareto-Optimal Solutions. These solutions form a set of trade-off solutions, where each solution optimizes the various objective functions to varying degrees.

Emergency resource scheduling is considered a multi-objective optimization problem. The existing solution methods for the models mainly include two types: one is traditional optimization methods, such as the linear weighting method, ideal point method, goal programming method, and heuristic algorithms. For instance, Wang employed an enhanced immune algorithm to develop a multi – objective optimization model for logistics and rescue resources in the South China Sea [20]. Sun introduced an emergency material scheduling model for multiple – storage – point and disaster – area scenarios, applying an improved genetic algorithm to jointly optimize scheduling time and material storage [21]. For small-scale problems, the solution effect of these traditional optimization methods is relatively ideal; the other type is intelligent optimization algorithms, which mainly include swarm intelligence algorithms and multi-objective genetic algorithms. When it comes to larger-scale problems, the solution effect can drop sharply. Although intelligent optimization algorithms cannot guarantee the optimal solution to the problem, they have a broader range of applicability and are not highly dependent on the initial solution. Early classic swarm intelligence algorithms mainly include ant colony optimization [22] and particle swarm optimization [23], Subsequently, new intelligent algorithms have been proposed, such as Bacterial Foraging Optimization Algorithm [24], Firefly Algorithm [25], and Beetle Antennae Search Algorithm [26]. For instance, Yang Bing proposed an improved ant-colony algorithm for rescue-path planning [27]. Xu proposed a hybrid Gaussian quantum particle swarm optimization and adaptive genetic algorithm (HGQPSO-AGA) to solve the job shop scheduling problem [28]. While traditional optimization methods and swarm intelligence algorithms have been applied to emergency resource scheduling, multi-objective genetic algorithms (MOGAs) have emerged as particularly effective for balancing conflicting objectives. Among these, the Non-dominated Sorting Genetic Algorithm II (NSGA-II) has gained prominence due to its two core mechanisms: Non-dominated sorting to hierarchically classify solutions based on Pareto dominance. Crowding distance calculation to maintain solution diversity in the objective space.

The superiority of NSGA-II is evidenced in emergency resource studies. For instance, Li optimized emergency material delivery routes in Hubei using NSGA-II, demonstrating its capability to handle integer programming models [29]. Ye constructed a hierarchical emergency resource scheduling model and solved it using an improved NSGA-II algorithm [30]. Liu constructed a risk informed multi-objective optimization model using the fast elitist non dominated sorting genetic algorithm (NSGA-II) [31].

The proposed model is a multi-objective mixed-integer programming formulation that requires balancing multiple objective functions and constraints, resulting in high computational complexity. This study employs the NSGA-II algorithm as the solution methodology due to its demonstrated advantages in computational efficiency and uniform distribution of solution sets when addressing multi-objective optimization problems, enabling effective identification of Pareto optimal solution sets. By establishing a multi-objective optimization framework that integrates timeliness, cost-effectiveness, and fairness, decision-makers can dynamically weigh competing objectives against practical constraints. Leveraging multi-criteria evaluation or priority ranking mechanisms, they can select the optimal resource allocation strategy from the Pareto solution set that aligns with the evolving disaster scenario and operational requirements.

## 2. Construction and solution of emergency resource dispatch models

**Fig 1:**
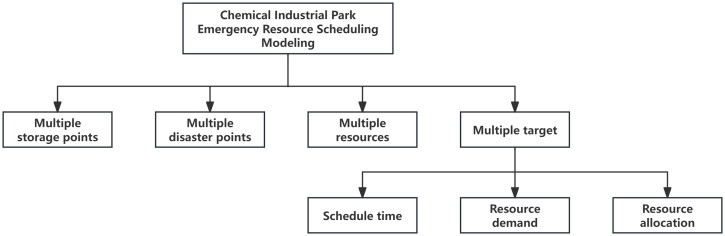
Analysis of elements of emergency resource scheduling model in chemical industry park.

The research objective of this section is how to optimize resource scheduling to shorten the time for emergency resources to reach the demand point, maximize the actual supply rate to meet each affected point, and improve the fairness of resource allocation under the multi-affected point accident in the chemical park by resolving the imbalance between resource supply and demand and the existence of the minimum demand satisfaction target. This study focuses on the analysis of the objective function and optimization conditions in the mathematical model, on the basis of which the emergency resource dispatch model for hazardous chemical accidents in chemical parks is constructed, and the elemental analysis of the model is shown in Fig 1.

### 2.1. Emergency resource dispatch modelling

#### 2.1.1. Objective function.

(1) Minimum time for emergency resources to reach the disaster site

Given the high risks of cascading failures in chemical accidents, delayed resource delivery may escalate casualties. We thus prioritize minimizing total emergency response time. Its calculation formula is shown in (2−1) [32].


$$\begin{array}{c} minT=\sum_{i=1}^{n}\left(T_{j}C_{ij}+t_{ij}G_{ij}\right) \end{array}$$ (2-1)

Transport activation‌: $C_{ij}\in \left\{\text{0},\text{1}\right\}$ indicates resource dispatch from depot j to disaster point i;

Vehicle allocation‌: $C_{ij}=\left[\Sigma_{m=1}^{u}b_{m}X_{ijm}\right]$, where $X_{ijm}$ is the quantity of resource m, and $b_{m}$ denotes its space occupancy per vehicle.

This integrates two critical time components: vehicle loading time ($T_{j}C_{ij}$) proportional to dispatched vehicles, and transportation duration ($t_{ij}G_{ij}$) dependent on route selection.

(2) Maximize Actual Demand Satisfaction Rate

To enhance resource utilization and ensure critical needs are prioritized, the model maximizes the actual demand satisfaction rate (Z) across all disaster points. This rate is calculated as the ratio of the total resources delivered ($a_{im}$) to the demand ($d_{im}$) for each resource type m at disaster point i, expressed by:


$$\begin{array}{c} maxZ=\sum_{i\in I}\sum_{m\in M}\frac{a_{\text{im}}}{d_{im}} \end{array}$$ (2-2)

where $a_{im}=\sum_{j\in J}X_{\text{ijm}}$ represents the total quantity of resource mtransported to disaster point i from all storage points j. This objective ensures efficient allocation while minimizing resource waste [33].

(3) Minimize Resource Allocation Inequality

To ensure balanced resource distribution across disaster points, the model minimizes the standard deviation (S) of the demand satisfaction rates ($Z_{im}$) for all resources and locations. The fairness objective is defined as:


$$minS=\sqrt{\frac{\sum_{i\in I}\sum_{m\in M}\left(Z_{im}-\overset{―}{Z}\right)}{I\times M-1}}$$ (2-3)

where $Z_{im}$=$a_{im}/d_{im}$ is the satisfaction rate of resource *m* at disaster point *i*, and $\overset{―}{Z}$is the average satisfaction rate across all points and resources. Minimizing *S* reduces disparities in resource allocation, preventing over-supply in some areas while others receive only minimal support [24].

#### 2.1.2. Constraints.

The model incorporates the following critical constraints to ensure feasibility and practical relevance:

(1) Resource capacity limits:

$$\begin{array}{c} maxZ=\sum_{i\in I}\sum_{m\in M}\frac{a_{\text{im}}}{d_{im}} \end{array}$$ (2-4)

The total resources allocated from storage point *j* for type *m* must not exceed its inventory$Q_{jm}$.

(2) Demand fulfillment:

$$a_{im}\leq d_{im},\forall i\in I,\forall m\in M$$ (2-5)

The quantity of resource *m* delivered to disaster point *i* ($a_{im}=\Sigma_{j}X_{ijm}$) cannot surpass its demand$d_{im}$.

(3) Minimum supply guarantee:

$$\begin{array}{c} \sum_{j\in J}X_{ijm}\geq \lambda d_{im},\forall i\in I,\forall m\in M \end{array}$$ (2-6)

At leastλ of the demand for each resource must be met to ensure baseline emergency response.

(4) Non-negative integers:

$$X_{ijm}\in Z_{+}$$ (2-7)

Resource quantities are discrete units

#### 2.1.3. Model assumption.

This paper proposes the following hypotheses based on the realistic environment of emergency resource dispatch for hazardous chemical accidents in chemical parks:

(1) The distance from each storage point to the disaster site is known;
(2) The resources between the storage points are independent of each other and there is no coordination and replenishment of resources;
(3) The space factor of the transport vehicle occupied for each type of resource is known;
(4) The time for loading resources in each vehicle is known;
(5) The speed at which the transport vehicle transports the emergency resources is known.

#### 2.1.4. Description of symbols and variables.

Based on the selected objective and constraint functions, the symbols involved in the model and their meanings are shown in Table 1.

**Table 1:** Symbolic meaning table.

Notation	Sense
I	Aggregation of affected areas{1,2...i}
J	Collection of emergency resource repositories{1,2...j}
M	Aggregation of types of emergency resources{1,2...m}
$d_{im}$	Demand for resources m in affected region i
z	Rate of supply of actual demand for resources at the affected site
$a_{im}$	Total resources transported to disaster point i
$X_{ijm}$	Quantity of the m-th resource transported from storage point j to affected point i
$T_{j}$	Lead time per vehicle to load resources at the storage site
$C_{ij}$	Number of vehicles required to transport resources from storage point j to disaster point i
$t_{ij}$	Travel time from storage point jto affected point i
$G_{ij}$	Whether to transport resources from storage point j to affected point i
$b_{m}$	Space factor per piece per transport vehicle for type m resources
S	Standard deviation of the rate of supply of real demand for goods
$Q_{jm}$	Storage of the m-th resource in storage pointj
λ	Minimum needs fulfilment rate

#### 2.1.5. Model formulation.

Building upon the objective functions and constraints defined in 2.1.1Objective function and 2.1.2 Constraints, this study synthesizes a multi-objective mixed-integer programming model for emergency resource scheduling in chemical industrial parks. The model integrates dynamic demands from multiple disaster points, coordinated supply from multiple storage points, and coupling constraints of multiple resource types. The formulation emphasizes three conflicting objectives: transportation efficiency, demand coverage, and allocation fairness, under practical operational limitations.

Key Components of the Model:

(1) Objective Functions (as defined in 2.1.1Objective function):
  - Minimize total transportation time (*T*, Eq. 2−1).
  - Maximize actual demand satisfaction rate (*Z*, Eq. 2−2).
  - Minimize resource allocation inequality (*S*, Eq. 2–3).
(2) Constraints (as defined in 2.1.2 Constraints):
  - Resource capacity limits (Eq. 2–4).
  - Demand fulfillment bounds (Eq. 2–5).
  - Minimum supply guarantee (Eq. 2–6).
  - Non-negative integer constraints (Eq. 2–7).

Model Synthesis and Significance:

The proposed framework addresses the trade-offs inherent in multi-disaster scenarios, where optimizing one objective may compromise others. By coupling these objectives with realistic constraints, the model enables decision-makers to explore Pareto-optimal solutions through the NSGA-II algorithm (2.2 Model solving based on NSGA-II algorithm). This approach ensures flexibility in balancing priorities such as rapid response (T), high demand coverage (Z), and equitable distribution (S), tailored to evolving emergency conditions.

### 2.2 Model solving based on NSGA-II algorithm

#### 2.2.1 Algorithm selection criteria.

In this study, a multi-objective mixed integer programming model is constructed, which contains many variables and constraints and increases the complexity of the solving process. The model considers multiple decision-making objectives at the same time, but a single optimal solution may not be feasible because there may be conflicts between them. As an effective method to solve multi-objective optimization problems, NSGA-II algorithm is highly praised for its low computational complexity, efficient running speed and good solution set distribution [34]. The algorithm can provide a series of Pareto non-inferior solutions and a variety of feasible schemes for decision makers in emergency resource scheduling, so that they can make choices according to specific conditions and preferences.

#### 2.2.2 NSGA-II Algorithm Solution and Analysis.

The NSGA-II algorithm is employed to solve the proposed multi-objective emergency resource scheduling model. Its implementation involves the following key steps, tailored to address the model’s complexity and ensure Pareto-optimal solution generation:

(1) Chromosome

Encoding and Initialization Each chromosome represents a candidate solution, encoded as a matrix$X=[X_{ijm}]$, where $X_{ijm}$ denotes the quantity of resource *m* dispatched from storage point *j* to disaster point *i*. The initial population is generated by randomly assigning integer values to $X_{ijm}$ under the constraints of Equations 2–4 to –2–7.

(2) Fitness Evaluation

The three objective functions (T,Z,S) defined in 2.1.1Objective function are calculated for each solution. Non-dominated sorting is then applied to classify solutions into hierarchical Pareto fronts based on dominance relationships [35].

(3) Crowding Distance Calculation

To maintain diversity in the objective space, crowding distance is computed for solutions within each front. This metric quantifies the density of solutions surrounding a given individual, prioritizing isolated solutions during selection.

(4) Genetic Operators
  - Crossover: Simulated binary crossover (SBX) is applied to parent solutions, ensuring offspring inherit feasible resource allocation patterns.
  - Mutation: Polynomial mutation introduces controlled variability to explore unvisited regions of the solution space while adhering to integer constraints.
(5) Elitist Strategy

A combined population of parents and offspring is sorted into fronts, and the top Nsolutions (where N is the population size) are retained based on non-dominated rank and crowding distance. This ensures convergence toward the Pareto front while preserving diversity.

(6) Termination Criteria The algorithm terminates when either:
  - A predefined maximum number of generations is reached.
  - The hypervolume indicator stabilizes (relative change < 1% over 50 generations).
(7) Pareto Front Analysis The final Pareto front is analyzed to identify trade-offs among objectives. Endpoint solutions are extracted, and convergence trends are visualized to guide decision-making (see 3.2 Algorithm comparison).

**Fig 2:**
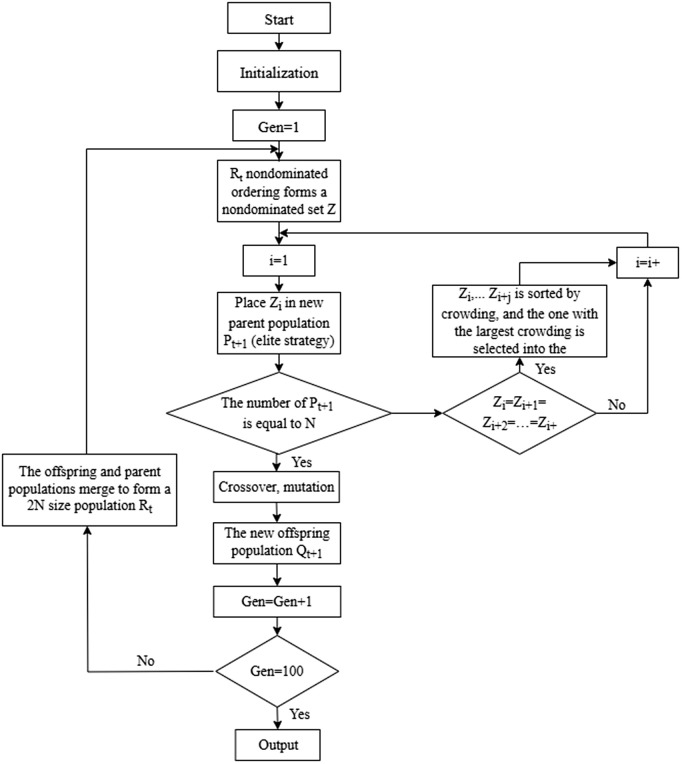
Pareto frontier solution solving process.

In summary, the solution process of the emergency resource scheduling model based on NSGA- II is shown in Fig 2.

By calculation, a set of Pareto optimal solutions that achieve the best balance among multiple objective functions is obtained. The resource scheduling scheme which reaches the extreme value on a certain objective function is selected, the level of the scheme on the other objective functions is analyzed. Furthermore, by depicting and analyzing the convergence trends of the values of each objective function in all Pareto front solutions, the relationships between the various objective functions in the emergency resource scheduling model are clarified.

#### 2.2.3. Multi-objective decision-making scheme selection.

The Pareto front derived from the NSGA-II algorithm provides a set of non-dominated solutions, each representing a unique trade-off among the three objectives: transportation time minimization (T), demand satisfaction maximization (Z), and equity optimization (S) [36]. To facilitate practical decision-making, a systematic framework is proposed to select the optimal emergency resource allocation plan based on decision-maker preferences and operational constraints.

Key Steps:

(1) Loss Metric Definition:

For each solution w in the Pareto front, normalized loss metrics are calculated to quantify deviations from the ideal values of each objective:

Transportation time loss:


$$LT_{i}=\frac{T_{w,1}-minT_{1}}{maxT_{1}-minT_{1}}$$ (2-8)

Demand satisfaction loss:


$$LZ_{i}=\frac{maxZ_{2}-Z_{w,2}}{maxZ_{2}-minZ_{2}}$$ (2-9)

Equity loss:


$$LS_{i}=\frac{S_{w,3}-minS_{3}}{maxS_{3}-minS_{3}}$$ (2-10)

Here, $T_{min}$,$Z_{max}$, and $S_{min}$denote the optimal values for each objective in the Pareto front.

(2) Weight Set Generation Steps:
  1) Basic Step Size Setting: Divide the weight interval into steps of 0.1 to balance computational efficiency and preference granularity (too fine steps increase redundant calculations, while too coarse steps may omit critical preferences).
  2) Hierarchical Generation: Extreme Preference Groups (6 groups): Single-objective weights ≥ 0.7, simulating emergency scenarios such as “time-priority” or “coverage-priority”; Two-Objective Balance Groups (15 groups): Both-objective weights ≥ 0.3, covering collaborative requirements such as “time-coverage” or “coverage-fairness”; Three-objective balanced groups (20 groups): weights are uniformly distributed, including combinations such as equal division and approximate balance, suitable for comprehensive decision-making during the stable phase of an incident.
  3) Validation and supplementation: combinations violating non-negativity are excluded, and special balanced weights are supplemented to ensure coverage completeness, ultimately forming 41 groups.
(3) Preference-Based Filtering:

Decision-makers specify tolerance thresholds for each loss metric based on scenario priorities. Solutions violating any threshold are eliminated, and the remaining candidates are ranked by weighted or lexicographic criteria.

(4) Dynamic Weight Assignment

For scenarios requiring balanced trade-offs, weights (𝑤𝑇, 𝑤𝑍, 𝑤𝑆) are assigned to the loss metrics, and solutions are evaluated using a composite score:


$${Score}_{w}=w_{T}\cdot {LT}_{w}+w_{Z}\cdot {LZ}_{w}+w_{S}\cdot {LS}_{w}$$

The solution with the minimum score is selected as optimal.

## 3 Case analysis

### 3.1 Background of chemical industrial park

The park is divided into four areas, area 1 is about 231 hectares, area 2 is about 246 hectares, area 3 is about 101 hectares, and area 4 is about 22 hectares. According to the “Map for Seismic Parameter regionalization of a certain Province” compiled and published by the Seismological Bureau and the Construction Department of the province, the seismic intensity of the park is VI-degree area and the site category is Class II. Due to the particularity of the location of the chemical park, it is assumed that an earthquake occurred in the area, which led to the simultaneous destruction of multiple tank areas in the chemical park and accidents at multiple disaster points.

### 3.2 Algorithm comparison

To test the performance of the improved multi-objective gray wolf optimization algorithm, three test functions, ZDT2, ZDT3 and ZDT6, were selected. The performance and stability of the NSGA-II algorithm are comprehensively judged by the evaluation metrics of hypervolume (HV), independent solution ratio (R independent), generation distance (GD), inverse generation distance (IGD) and standard deviation (SD).

The HV value measures the volume of the target space surrounded by the non-dominated solution set and the reference point. Larger values indicate that the solution set is closer to the true Pareto front and the diversity is better. R independence reflects the diversity of the solution set. The larger the value, the greater the diversity of the solution set, and the wider the search range of the algorithm.GD measures the proximity of the solution set to the true frontier, and the smaller the value, the closer the solution set is to the true frontier. IGD evaluates the homogeneity of the solution set, i.e., the coverage of the true frontier; the smaller the value, the more comprehensive the coverage of the true frontier and the more uniform the distribution of the solution set.SD reflects the degree of discretization of the results of the algorithm in multiple runs, which embodies the stability of the results. SD reflects the degree of dispersion of the algorithm’s multiple runs, reflecting the stability of the results.


$$ZDT2=\left\{ \;\;\;\begin{array}{c} f_{1}(x)=x_{1}\\ f_{2}(x)=g(x)\left(1-{\left(\frac{f_{1}}{g(x)}\right)}^{2}\right)s.t.x_{i}\in [0,1],i=1,2,\ldots ,25\\ g(x)=1+\frac{9}{n-1}\sum_{i=2}^{n}x_{i} \end{array}\right.$$

$$ZDT3=\left\{ \;\;\;\;\begin{array}{c} f_{1}(x)=x_{1}\\ f_{2}(x)=g(x)\left(1-\sqrt{\frac{f_{1}}{g(x)}}-\frac{f_{1}}{g(x)}sin(10\pi f_{1})\right)s.t.\\ g(x)=1+\frac{9}{n-1}\sum_{i=2}^{n}x_{i} \end{array}\right.$$

Here,$x_{i}\in [0,1],i=1,2,...,25$

Under the same conditions, NSGA-II was compared with the MOGWO algorithm and the weighted method through performance testing. The results based on the above evaluation indicators are shown in Table 2.

**Table 2:** The results of each test function.

Test function	Evaluation indicators	Weighted method	MOGWO	NSGA-II	Improvement
**ZDT2**	Hypervolume	0.794617	0.792258	0.786441	0.51%
	GD	0.0090322	0.008224	0.007467	6.54%
	IGD	0.003698	0.004251	0.003336	27.79%
	R_independent_	59.65%	60.75%	65.74%	6.46%
	SD	0.011453	0.010014	0.000746	92.55%
**ZDT3**	Hypervolume	0.567636	0.568917	0.578468	1.68%
	GD	0.012317	0.010214	0.009758	4.46%
	IGD	0.013241	0.011356	0.0085481	27.73%
	R_independent_	57.24%	57.83%	62.57%	8.20%
	SD	0.042841	0.014574	0.009457	35.11%

From the experimental data, it can be observed that NSGA-II outperforms the weighted method and MOGWO in several key metrics, demonstrating significant performance advantages and stability. NSGA-II’s HV and GD outperform other algorithms. In the test function, compared to the MOGWO algorithm, they improved by 7.70% and 6.54%, respectively. This indicates that its solution set is closer to the true Pareto front, and the algorithm demonstrates excellent convergence. Similarly, the IGD of R-independent NSGA-II outperforms other algorithms, with improvements of 8.20% and 35.66%, respectively. This indicates that the solution set covers a broader range and exhibits greater diversity. In terms of algorithm stability, the SD of NSGA-II is significantly better than other algorithms. Especially in the ZDT6 function, the SD is 97.70% higher than the MOGWO algorithm, verifying the algorithm’s strong robustness for complex problems.

**Fig 3:**
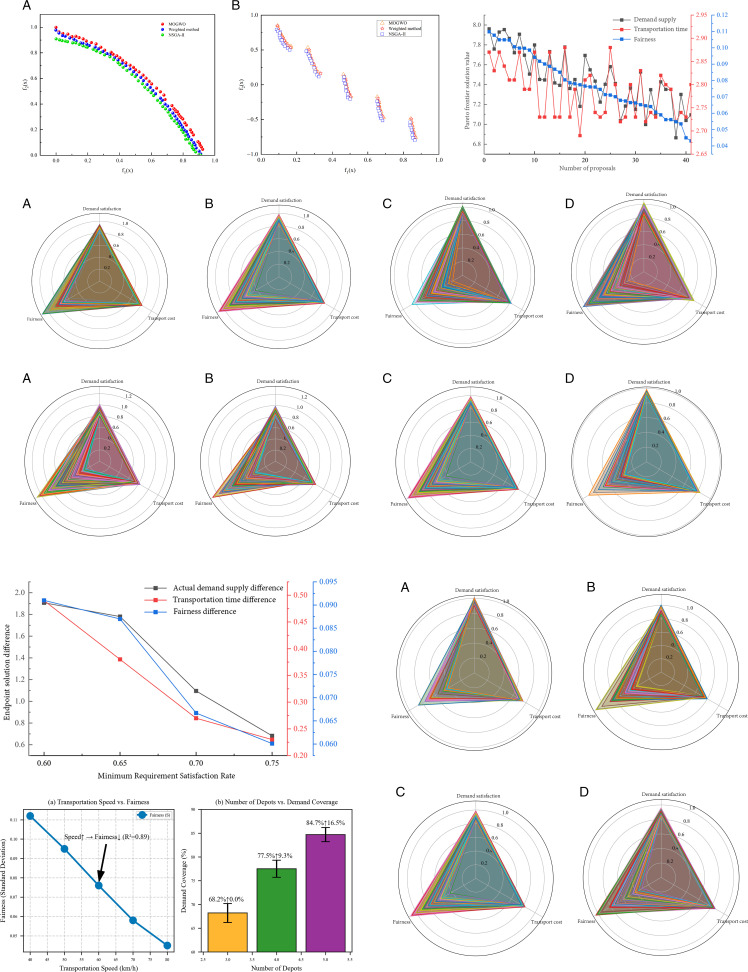
Experimental results and sensitivity analysis.

The experimental results of the Pareto frontier for each test function are shown in Fig 3. It is clear that the solution set of the NSGA-II algorithm is of higher quality and closer to the true frontier.

### 3.3 Case study

#### 3.3.1 Case data.

Under this incident scenario, it is assumed that the affected areas are the tank areas within three districts, namely I_1_ (District One), I_2_ (District Three), and I_3_ (District Four). There are a total of four emergency resource storage points around the park, which are J_1_, J_2_, J_3_, and J_4_. The types of resources include three major categories: firefighting and rescue resources, medical rescue resources, and leak control resources. Due to the large number of detailed resource types, for the convenience of analysis, representative resources are selected, namely foam fire extinguishing agent, rescue stretcher, and sealing bucket, which are M_1_, M_2_, and M_3_ respectively.

(1) Resource Demand and Spatial Coefficients

The demand for each resource type at disaster points and their spatial coefficients (resource volume per vehicle) are provided in Table 3. Spatial coefficients are calculated based on the ratio of resource volume to standard vehicle capacity (13 m³).

**Table 3:** Resource Demand and Spatial Coefficients.

Disaster Area	Resource Type	Demand (t)	Spatial Coefficient
I_1_	M_1_	98	0.054
I_1_	M_2_	79	0.028
I_1_	M_3_	28	0.019
I_2_	M_1_	110	0.054
I_2_	M_2_	65	0.028
I_2_	M_3_	31	0.019
I_3_	M_1_	130	0.054
I_3_	M_2_	81	0.028
I_3_	M_3_	15	0.019

(2) Depot Resource Transportation Information

Loading times at depots and transportation times between depots and disaster points are listed in Table 4. Transportation time is calculated based on route distance and vehicle speed (60 km/h).

**Table 4:** Resource loading time(h).

Depot	Loading Time (h)	Transportation Time (h)
J_1_	0.08	I₁: 0.12, I₂: 0.05, I₃: 0.09
J_2_	0.09	I₁: 0.11, I₂: 0.12, I₃: 0.08
J_3_	0.10	I₁: 0.10, I₂: 0.08, I₃: 0.15
J_4_	0.06	I₁: 0.13, I₂: 0.07, I₃: 0.14

(3) Depot Resource Inventory and Minimum Supply

Minimum supply thresholds (λ = 0.8) and actual inventory levels for each resource type are detailed in Table 5.

**Table 5:** Depot resource inventory and minimum supply (t).

Resource Type	Depot	Minimum Supply	Inventory
M_1_	J_1_	78	85
M_1_	J_2_	67	84
M_1_	J_3_	63	66
M_1_	J_4_	22	63
M_2_	J_1_	88	90
M_2_	J_2_	67	84
M_2_	J_3_	52	75
M_2_	J_4_	25	60
M_3_	J_1_	104	110
M_3_	J_2_	64	81
M_3_	J_3_	65	78
M_3_	J_4_	12	78

#### 3.3.2 Analysis of solution results.

The NSGA-II algorithm generated 41 Pareto-optimal solutions. Key endpoint solutions and their trade-offs are summarized in Table 6. See Appendix S1 Table for complete explanation.

**Table 6:** The Pareto frontier solution of NSGA-Ⅱ.

Scheme Name	Demand supply	Transportation time	Fairness	Demand supply loss (%)	Transportation time loss (%)	Fairness loss (%)
#1	7.961	2.87	0.1098	0	66.666	100
#4	7.954	2.96	0.1049	0.682	100	92.696
#5	7.865	2.81	0.1047	8.729	44.444	92.343
#19	7.181	2.69	0.0774	91.275	0	51.373
#41	7.096	2.8	0.0431	78.917	40.741	0

By analyzing the endpoint values and convergence trends of the Pareto front solutions, the relationships between the demand supply rate, transportation time, and equity of resource allocation in emergency resource scheduling are determined. According to Table 6, the demand supply loss for the affected points in Plan #1 and Plan #4 are 0 and 0.682, respectively, indicating that under these two plans, the demand supply rate for various resources at each affected point is relatively high. However, the transportation time loss is as high as 100% and 92.696%, respectively, indicating a longer transportation time. In contrast, in Plan #19, the transportation time loss for various resources is 0, meaning the total transportation time for various resources is the shortest, but at this time, the demand satisfaction loss for each affected point reaches 91.275%, only maintaining a lower level of resource demand supply. In Plan #41, the equity loss of emergency resource allocation is 0, meaning that the resource allocation is the most balanced under this plan. However, the demand satisfaction loss is as high as 78.917%, indicating a lower actual rate of resource demand satisfaction.

The distribution and convergence trend of the Pareto front solutions are shown in Fig 3. It can be observed that there is a clear trade-off relationship between the demand supply objective and the transportation time objective, meaning that increasing the actual demand supply rate of resources requires a certain sacrifice in transportation time; there is a certain trade-off relationship between transportation time and equity, meaning that in some cases, an increase in equity may lead to an increase in transportation time. Since the number of obtained Pareto front solutions is relatively large, decision-makers can choose an appropriate resource scheduling plan based on specific accident conditions or their own preferences.

To further analyze the connections between multiple objective functions, data analysis is conducted on the resource scheduling plans under optimal solutions for individual objective functions. The plan with the maximum demand supply is #1, the one with the minimum transportation time is #19, and the one with the best equity is #41, as shown in Table 7.

**Table 7:** The function value of the single objective optimal solution.

Optimization objectives	Scheme name		
	#1	#19	#41
Demand supply	7.961	7.181	7.096
Transportation time	2.87	2.69	2.8
Fairness	0.1098	0.0774	0.0431

From Table 7, it can be observed that Plan #1 performs the best in terms of demand supply, reaching 7.961, but compared to Plans #19 and #41, it has poorer performance in transportation time and resource allocation equity. This indicates that although Plan #1 can meet the demand to the greatest extent, it comes at the cost of increased transportation time and uneven resource allocation. On the other hand, Plan #41 shows the best performance in equity, with a value of 0.023, but its performance in demand satisfaction and transportation time is relatively poor. Plan #19, however, provides the shortest transportation time, which is 2.69, but it has a lower demand supply compared to Plan #1 and a poorer equity compared to Plan #41.

#### 3.3.3 Decision scheme selection.

The proposed multi-objective decision-making method based on the NSGA-II algorithm is linked to the final weight selection through the Pareto optimal solution set, which bridges the gap between the algorithm’s output and decision preferences. The 41 non-dominated solutions generated by NSGA-II cover all possible trade-off relationships among transportation time, demand coverage, and allocation fairness, while weight selection represents the decision-maker’s quantitative expression of priority rankings for different objectives based on actual emergency scenarios. Specifically, first, the deviation between each solution and the ideal values of each objective is calculated using a normalized loss metric. Then, combined with the weights set by the decision-maker, a comprehensive loss scoring model is constructed. Finally, the solution with the smallest comprehensive loss is selected from the solution set. This association mechanism retains the algorithm’s global optimization capability for multi-objective conflicts while incorporating the decision-maker’s subjective preferences through weights. This ensures that the final selected scheduling solution reflects both scientifically optimized results and adapts to dynamically changing emergency needs. For example, during the initial phase of an accident, when transportation time is prioritized, a solution with a high time weight is selected; during the stabilization phase, when fairness is emphasized, the weights are adjusted to match solutions with balanced distribution.

It can be seen that decision-makers can choose a satisfactory resource scheduling plan based on their own tolerance for demand supply loss, transportation time loss, and equity loss, as shown in Table 8. When the decision-maker’s tolerance for demand supply loss is 10%, then among the plans where demand supply loss is ≤ 10%, choose the plan with the smallest combined transportation time loss and equity loss, the result is Plan #5; when the decision-maker’s tolerance for transportation time loss is 10%, then among the plans where transportation time loss is ≤ 10%, choose the plan with the smallest combined actual demand loss and equity loss, the result is Plan #19; when the decision-maker’s tolerance for equity loss is 10%, then among the plans where equity loss is ≤ 10%, choose the plan with the smallest combined actual demand loss and transportation time loss, the result is Plan #40. The analysis method is the same for other levels of tolerance.

**Table 8:** Decision maker preference and scheme selection.

Decision maker preferences		Scheme selection	Actual losses		
			Demand supply loss/%	Transportation time loss/%	Fairness loss/%
Demand supply loss/%	10%	#5	8.729	44.444	93.343
	20%	#2	18.502	14.814	96.779
	30%	#20	24.391	44.444	50.232
	40%	#31	39.646	51.851	33.203
	50%	#12	46.877	14.814	70.401
Transportation time loss/%	10%	#19	91.725	0	51.373
	20%	#39	60.285	18.518	15.668
	30%	#39	60.285	18.518	15.668
	40%	#31	39.646	51.851	33.203
	50%	#35	48.589	48.148	23.858
Fairness loss/%	10%	#40	84.129	14.814	2.836
	20%	#39	60.285	18.518	15.668
	30%	#39	60.285	18.518	15.668
	40%	#33	55.786	18.518	31.539
	50%	#24	50.918	18.518	42.481

Taking a special case as an example, when the decision-maker strives to achieve the most ideal situation for one of the resource demand supply rates, transportation time, and distribution equity, the corresponding emergency resource scheduling plans are Plans #1, #19, and #41, respectively. At this time, the corresponding quantities of emergency resource scheduling are shown in Tables 9–11. Similarly, decision-makers can choose other emergency resource scheduling quantity plans based on their tolerance for the loss of the objective function.

**Table 9:** Demand meets the maximum amount of emergency resources supplied.

Types of Resources	Disaster stricken sites	Storage point			
		J_1_	J_2_	J_3_	J_4_
M_1_	I_1_	25	20	11	15
	I_2_	26	23	22	24
	I_3_	21	27	24	23
M_2_	I_1_	15	26	15	22
	I_2_	12	8	18	24
	I_3_	21	14	24	3
M_3_	I_1_	12	12	0	4
	I_2_	22	3	1	3
	I_3_	8	0	6	1

**Table 10:** The quantity of emergency resource supply under the shortest transportation time.

Types of Resources	Disaster stricken sites	Storage point			
		J_1_	J_2_	J_3_	J_4_
M_1_	I_1_	16	22	9	24
	I_2_	19	21	16	22
	I_3_	28	27	24	15
M_2_	I_1_	13	22	14	22
	I_2_	10	23	2	16
	I_3_	8	27	13	20
M_3_	I_1_	13	4	6	3
	I_2_	19	3	1	3
	I_3_	2	3	5	1

**Table 11:** Quantity of emergency resource supply under optimal resource allocation.

Types of Resources	Disaster stricken sites	Storage point			
		J_1_	J_2_	J_3_	J_4_
M_1_	I_1_	16	21	20	24
	I_2_	19	18	22	22
	I_3_	28	27	24	15
M_2_	I_1_	13	22	15	14
	I_2_	10	10	17	16
	I_3_	22	12	13	20
M_3_	I_1_	13	0	6	3
	I_2_	19	3	1	3
	I_3_	2	3	5	1

Comparing the three transportation plans mentioned above, the quantities of resources scheduled for the three affected points under the condition of maximizing the resource demand supply rate are calculated to be 177 t, 186 t, and 172 t, totaling 535 t. In contrast, the quantities of resources scheduled under the plans with the shortest transportation time and the most equitable resource distribution are 496 t and 499 t, respectively. It can be observed that to meet the resource demand supply rate, the quantity of emergency resources in the scheduling plan will significantly increase.

### 3.4 Sensitivity analysis

To identify the impact of different parameters on the output results of the emergency resource scheduling model, enhance the efficiency of emergency resource scheduling, and assist decision-makers in understanding the trends of model results under different conditions and make reasonable decisions, this section conducts a sensitivity analysis of the emergency resource scheduling model through simulation, selecting three parameters: resource storage volume, minimum demand satisfaction rate, and the resource demand of the affected points.

#### 3.4.1 Sensitivity analysis of resource storage capacity.

Keeping other parameters constant, the storage of emergency resources at the storage points is changed to 0.9 times, 1.1 times, and 1.2 times of the original, respectively. The radar chart of the optimal solutions of the emergency resource scheduling plan generated from the Pareto front is shown in Fig 3.

Based on the simulation results in Fig 3, it can be observed that under the initial set storage quantity, there are 41 solutions on the Pareto front. When the storage quantity is adjusted to 0.9 times, 1.1 times, and 1.2 times the initial value, the number of plans is 34, 46, and 57, respectively. This indicates that when comprehensively considering the transportation time cost, actual demand supply rate, and equity of resource distribution, there is a positive correlation between the resource storage quantity at the storage points and the number of emergency resource scheduling plans, meaning that increasing the resource storage quantity will yield more feasible emergency resource scheduling plans.

#### 3.4.2 Sensitivity analysis of minimum requirement satisfaction rate.

With all other parameters remaining constant, the minimum resource requirement satisfaction rates for each disaster point are set to 0.6, 0.65, 0.7, and 0.75, respectively. The radar chart of the optimal emergency resource scheduling plan based on the Pareto frontier is shown in Fig 3.

According to Fig 3, when the minimum resource requirement satisfaction rate is 0.6 and 0.65, the number of Pareto optimal solutions is 76 and 64, respectively, which is more than when the minimum resource requirement satisfaction rate is 0.7 and 0.75. This indicates that when the demand for resources at each disaster point is relatively low, there are more implementable resource scheduling plans. However, from the distribution area of the radar chart, it can be observed that as the minimum resource requirement satisfaction rate increases, the area occupied by each solution increases, indicating that the number of scheduling plans is decreasing, but the plans are more optimal in various aspects.

Table 12 and Fig 3 illustrate the range of differences in the optimal endpoint solutions under different minimum resource requirement rates. As the minimum demand satisfaction rate increases, the difference between the upper and lower endpoint values of the objective function in the Pareto optimal solutions is also decreasing. Taking the demand satisfaction as an example, when λ = 0.6, the difference in the objective function S between the upper and lower endpoint values of the Pareto frontier solution is 1.9074. When λ = 0.65, the difference is 1.7804, which is a reduction of 0.127 compared to when λ = 0.6. This indicates that the adjustable range of emergency resource scheduling is narrowing, and the differences in emergency resource demand satisfaction among various disaster points are diminishing, which plays a positive role in balancing the fairness of resource distribution. Therefore, when formulating a plan, it is necessary to set a reasonable minimum demand satisfaction rate based on the disaster situation, to make the scheduling plan relatively reasonable, and to ensure the smooth progress of emergency rescue work.

**Table 12:** The difference between the end points of the objective function for different minimum requirement satisfaction rates.

Minimum Requirement Satisfaction Rate	Actual demand supply difference	Transportation time difference	Fairness difference
0.6	1.9074	0.49	0.091
0.65	1.7804	0.38	0.087
0.7	1.0956	0.27	0.0667
0.75	0.6821	0.23	0.0601

#### 3.4.3 Sensitivity analysis of demand quantity.

With all other parameters held constant, the predicted disaster demand is set to 0.8, 0.9, and 1.1 times the initial value, respectively. The radar chart of the optimal emergency resource scheduling plan based on the Pareto frontier is shown in Fig 3.

From Fig 3, it can be seen that when there is a certain change in the resource demand of the disaster points, it is intuitively apparent that the number of Pareto optimal solutions decreases rapidly, indicating that the resource demand of the disaster points is the most significant factor affecting the fluctuation in the number of feasible resource scheduling plans. Clearly calculating the average values of resource supply and transportation costs for the scheduling plans obtained under the four demand conditions, the average values of resource supply are 7.773, 7.491, 7.435, and 7.199, and the average values of transportation time are 2.54, 2.63, 2.79, and 2.88. It can be observed that as the resource demand of the disaster points increases, the average levels of resource supply and transportation time in the scheduling plans are decreasing, indicating that an increase in the number of resources will lower the level of the resource scheduling plans.

Considering Fig 3, when there are certain changes in resource storage, the minimum requirement satisfaction rate, and the resource demand of the disaster points, the changes in demand satisfaction and transportation time in each individual radar chart for emergency resource scheduling plans are not significant. The main difference between the plans is reflected in the fairness of resource allocation, which also demonstrates the necessity of considering the objective function of resource allocation fairness in the emergency resource scheduling model.

#### 3.4.4 Sensitivity Analysis of Operational Parameters.

In addition to the supply-demand parameters analyzed above (resource inventory, minimum requirement satisfaction rate, and demand quantity), emergency resource scheduling in chemical parks is also influenced by operational parameters, such as transportation speed and depot quantity. These factors directly impact the trade-offs among transportation efficiency, demand coverage, and fairness. This section evaluates their effects on the proposed model.

The sensitivity analysis reveals that increasing transportation speed (40–80 km/h) reduces total transportation time by 28% (from 3.2 h to 2.3 h) but degrades fairness (S increases by 23%, from 0.045 to 0.112) due to imbalanced resource allocation, while marginally lowering demand satisfaction (Z) by 6% (Fig 3). Expanding depot quantity from 4 to 5 improves demand coverage by 9.3% (77.5% to 84.7%) but raises transportation time by 14% (2.8 h to 3.2 h) and slightly enhances fairness (S reduced by 6%) through flexible distribution (Fig 3). These findings highlight critical trade-offs: faster speeds prioritize time efficiency over equity, whereas additional depots boost coverage at the cost of coordination delays, guiding tailored strategies for time-critical versus large-scale disasters.

## 4 Conclusion

This paper investigates the optimization of emergency resource allocation in chemical industrial parks. Based on the NSGA-II algorithm, a multi-objective mixed-integer programming model specifically tailored for chemical industrial parks is constructed, integrating three core objectives: minimizing total transportation time, maximizing actual demand satisfaction rate, and minimizing resource allocation inequality. By explicitly incorporating fairness as a key optimization objective and considering the unique characteristics of accidents in chemical industrial parks, this model fills a gap in existing research. Constraints such as resource capacity limits, demand fulfillment ranges, and minimum supply guarantees ensure the model’s practical applicability. In a case study, performance comparison experiments validated NSGA-II as an effective solution method. Comparisons with the weighted method and MOGWO algorithm demonstrated that NSGA-II outperforms these methods in key metrics such as hypervolume (HV), generation distance (GD), inverse generation distance (IGD), and stability (SD). This confirms its excellent convergence, solution diversity, and robustness, making it highly suitable for addressing complex multi-objective scheduling problems in chemical industrial park scenarios. Based on this, the algorithm was subsequently applied to a real-world scenario. A case study involving three disaster points and four resource storage points in a chemical industrial park revealed the inherent trade-offs between objectives: high demand satisfaction often comes at the cost of increased transportation time, while optimal fairness may lead to reduced satisfaction rates. To address this issue, a multi-criteria decision-making framework for scheme selection was established: normalized loss metrics were calculated for each Pareto optimal solution to quantify deviations from ideal values for transportation time, demand satisfaction, and fairness. Forty-one sets of weights were generated, covering extreme preferences, two-objective balance, and three-objective equilibrium, enabling decision-makers to screen solutions based on their tolerance thresholds for losses. The optimal solution was selected by minimizing the comprehensive weighted loss score, ensuring alignment with actual emergency priorities.

Finally, the study determined through sensitivity analysis that resource demand is the most critical factor influencing the number of feasible solutions; higher demand significantly reduces planning flexibility. The study found that resource storage capacity and minimum satisfaction rate are positively correlated with solution diversity, while fairness emerges as the key distinguishing factor between scheduling plans, emphasizing its necessity in emergency resource allocation. This study provides a systematic and scientific tool for emergency resource scheduling in chemical industrial parks, effectively balancing timeliness, demand coverage, and allocation fairness. It offers practical insights for enhancing emergency response capabilities and mitigating disaster losses in such high-risk environments.

To expand the application scope of the NSGA-II algorithm and scheduling model, we plan to deepen research in the following areas: (1) Actively seek collaboration with safety-related departments to integrate more real-world scenario data and introduce more representative test cases to further validate the applicability and stability of the algorithm and model. (2) Further integrating multi-agent collaborative scheduling mechanisms, as cross-departmental resource coordination has not been addressed, yet chemical plant accident rescue often requires multi-agent collaboration. Future research may incorporate game theory or distributed optimization methods to analyze conflicting objectives among different rescue entities, design collaborative decision-making mechanisms, and optimize the overall efficiency of resource allocation.

## Supporting information

S1 Table The Pareto frontier solution of NSGA-Ⅱ. (DOCX)
