# Supplementary material for: Research on multi-objective emergency resource scheduling optimization in chemical industrial parks
Source: PLoS One. 2025 Sep 30;20(9):e0332858. doi: 10.1371/journal.pone.0332858 (PMC12483207; doi:10.1371/journal.pone.0332858)
Supplement: S1 Table — (DOCX) [file pone.0332858.s001.docx]

**Supporting information**

**Table S1 The Pareto frontier solution of NSGA-Ⅱ**

| **Scheme Name** | **Demand supply** | **Transportation time** | **Fairness** | **Demand supply loss (%)** | **Transportation time loss (%)** | **Fairness loss (%)** |
| --- | --- | --- | --- | --- | --- | --- |
| #1 | 7.961 | 2.87 | 0.1098 | 0 | 66.666 | 100 |
| #2 | 7.758 | 2.73 | 0.1077 | 18.502 | 14.814 | 96.779 |
| #3 | 7.929 | 2.87 | 0.1049 | 2.962 | 66.666 | 92.701 |
| #4 | 7.954 | 2.96 | 0.1049 | 0.682 | 100 | 92.696 |
| #5 | 7.865 | 2.81 | 0.1047 | 8.729 | 44.444 | 92.343 |
| #6 | 7.721 | 2.81 | 0.1006 | 21.891 | 44.444 | 86.214 |
| #7 | 7.907 | 2.87 | 0.0998 | 4.941 | 66.666 | 85.029 |
| #8 | 7.696 | 2.79 | 0.0997 | 24.195 | 37.037 | 84.851 |
| #9 | 7.506 | 2.79 | 0.0986 | 41.577 | 37.037 | 83.139 |
| #10 | 7.799 | 2.87 | 0.0942 | 14.755 | 66.666 | 76.636 |
| #11 | 7.452 | 2.73 | 0.0918 | 46.456 | 14.814 | 73.065 |
| #12 | 7.447 | 2.73 | 0.0901 | 46.877 | 14.814 | 70.401 |
| #13 | 7.732 | 2.87 | 0.0887 | 20.899 | 66.666 | 68.418 |
| #14 | 7.417 | 2.73 | 0.0869 | 49.664 | 14.814 | 65.626 |
| #15 | 7.393 | 2.73 | 0.0851 | 51.823 | 14.814 | 62.966 |
| #16 | 7.778 | 2.88 | 0.0804 | 16.697 | 70.371 | 55.963 |
| #17 | 7.361 | 2.73 | 0.0786 | 54.745 | 14.814 | 53.177 |
| #18 | 7.451 | 2.79 | 0.078 | 46.594 | 37.037 | 52.378 |
| #19 | 7.181 | 2.69 | 0.0774 | 91.275 | 0 | 51.373 |
| #20 | 7.694 | 2.81 | 0.0766 | 24.391 | 44.444 | 50.232 |
| #21 | 7.549 | 2.82 | 0.0761 | 37.651 | 48.148 | 49.526 |
| #22 | 7.435 | 2.74 | 0.076 | 47.973 | 18.518 | 49.401 |
| #23 | 7.224 | 2.73 | 0.0746 | 67.2837 | 14.814 | 47.284 |
| #24 | 7.403 | 2.74 | 0.0714 | 50.918 | 18.518 | 42.481 |
| #25 | 7.581 | 2.88 | 0.0711 | 34.658 | 70.371 | 41.926 |
| #26 | 7.411 | 2.8 | 0.0701 | 50.183 | 40.741 | 40.551 |
| #27 | 7.036 | 2.72 | 0.0679 | 84.442 | 11.111 | 37.192 |
| #28 | 7.184 | 2.73 | 0.0675 | 70.923 | 14.814 | 36.554 |
| #29 | 7.365 | 2.8 | 0.0665 | 54.395 | 40.741 | 35.114 |
| #30 | 7.151 | 2.73 | 0.0663 | 73.999 | 14.814 | 34.828 |
| #31 | 7.527 | 2.83 | 0.0652 | 39.646 | 51.851 | 33.203 |
| #32 | 6.998 | 2.72 | 0.0646 | 87.822 | 11.111 | 32.198 |
| #33 | 7.349 | 2.74 | 0.0641 | 55.786 | 18.518 | 31.539 |
| #34 | 7.108 | 2.73 | 0.0607 | 77.855 | 14.814 | 26.345 |
| #35 | 7.428 | 2.82 | 0.059 | 48.589 | 48.148 | 23.858 |
| #36 | 7.352 | 2.83 | 0.0561 | 55.506 | 51.851 | 19.549 |
| #37 | 7.352 | 2.83 | 0.0561 | 55.506 | 51.851 | 19.549 |
| #38 | 6.865 | 2.72 | 0.0548 | 100 | 11.111 | 17.547 |
| #39 | 7.301 | 2.74 | 0.0535 | 60.285 | 18.518 | 15.66 |
| #40 | 7.039 | 2.73 | 0.045 | 84.129 | 14.814 | 2.836 |
| #41 | 7.096 | 2.8 | 0.0431 | 78.917 | 40.741 | 0 |
